# Supplementary material for: Are carbon emissions trading and green financial instruments synergistic? -Comprehensive quantitative research based on content analysis
Source: PLoS One. 2024 Mar 7;19(3):e0298601. doi: 10.1371/journal.pone.0298601 (PMC10919601; doi:10.1371/journal.pone.0298601)
Supplement: S1 Data — (ZIP) [file pone.0298601.s001.zip › Data/地级市Z_F工作报告.zip!/╣π╬≈/╣≡┴╓╩╨/└┤╫╘╣1⁄2╓┌║┼í╛┬φ┐╦╩2╛▌═°í┐_2018.docx]

各位代表：

　　现在，我代表市人民政府向大会作工作报告，请予审议，并请各位政协委员和列席会议的同志提出意见。

　　一、2017年工作回顾

　　过去的一年，面对经济持续下行和特大洪涝灾害双重考验，在自治区党委、政府和市委的领导下，我们主动适应经济发展新常态，全面落实新发展理念，坚持桂林国际旅游胜地建设“一本蓝图绘到底”，统筹推进稳增长、促改革、调结构、惠民生、防风险各项工作，保持了经济社会持续健康发展。初步预测，地区生产总值增长5.0%，组织财政收入增长7.0%，固定资产投资增长4.4%，社会消费品零售总额增长11.0%，城镇居民人均可支配收入增长8.0%，农村居民人均可支配收入增长11.0%，居民消费价格上涨1.6%，节能减排指标完成自治区下达任务。

　　—— 胜地魅力充分彰显。“央视春晚，桂林最美”享誉海内外，成功举办第十一届联合国世界旅游组织/亚太旅游协会旅游趋势与展望国际论坛、2017中国—东盟博览会旅游展、第七届桂林国际山水文化旅游节，2017环广西公路自行车世界巡回赛(桂林段)、桂林国际马拉松赛、漂流世界杯等国际赛事精彩纷呈，极大地提升了桂林知名度和美誉度。全年接待旅游总人数突破8000万人次，增长48.5%，旅游总消费达960亿元，增长50.5%;接待境外游客245万人次，增长6.0%，旅游外汇消费85亿元，增长9.0%。

　　—— 城市形象显著提升。桂阳文化旅游大道全面完工，漓江桥扩建、龙门大桥、香江立交等一批工程建成投入使用，是近年来基础设施项目竣工最多的一年。开展规模大、力度强、效果好的全国文明城市创建活动，解决了一大批市民反映强烈、长期想解决而没有解决的突出问题，城市面貌焕然一新。

　　—— 改革红利加速释放。系统推进83项改革任务、19项重点改革专题，旅游产业用地、导游管理体制改革走在全国前列，漓江管理体制改革、市级旅游资源整合取得重大突破，生态环境保护、文化发展、旅游转型升级等方面的实践和探索得到《人民日报》的高度关注。

　　—— 招大引强成果丰硕。成功引进华为、中兴、比亚迪、美亚迪、中联智创、释码大华等国内外知名高新技术企业，实现当年签约落地、当年开工建设，为重振桂林工业雄风注入了强劲动力。

　　—— 县域发展亮点突出。第三批16个新型城镇化示范乡镇“书记工程”基本完成，新型城镇化建设“桂林模式”受到区内外广泛关注。我市作为7个代表之一，在全国农村精神文明建设工作经验交流会作典型发言。全区“美丽广西·宜居乡村”工作现场会在我市召开，产业富民工作经验全区推广。脱贫攻坚取得阶段性成效，预计实现110个贫困村摘帽、7.8万贫困人口脱贫。荔浦、阳朔、灌阳成为广西科学发展十佳县，全州成为广西科学发展进步县，数量居全区之首。

　　一年来，我们主要做了以下工作：

　　(一)聚焦胜地建设，旅游发展水平实现新提升

　　坚持规划引领，统筹推进世界一流旅游目的地、全国生态文明建设示范区、全国旅游创新发展先行区、区域性文化旅游中心和国际交流重要平台建设，胜地建设中期目标全面实现，综合实力显著增强。实施“旅游+”行动，民宿乡居、体育休闲等新业态蓬勃发展，我市成为首批国家健康旅游示范基地，阳朔获评首批中国优秀国际乡村旅游目的地。实施旅游“双创”[1]行动，阳朔、兴安、龙胜、恭城、雁山全域旅游示范区建设稳步推进，灵川入围广西特色旅游名县创建县，阳朔成为全国首个基层标准化改革创新先行区创建县，“双创”工作保持全区领先。实施“领军企业培育”行动，引导旅发展、旅股份等进行资源整合、差异发展，打造桂林旅游旗舰企业。实施“旅游品质提升”行动，两江四湖(二期)连通水系水道建成通航，漓江城市段西岸慢行步道全面完工，两江四湖·象山景区晋升国家5A景区，阳朔西街晋升4A景区，兴安灵渠成为国家水利风景区，万达文化旅游城、独秀峰·王城景区5A提升等重大项目加快实施，桂林旅游产业研究院和旅游大数据中心挂牌成立，“厕所革命”桂林模式在全国推广。实施“旅游市场综合整治”行动，成立全区首支旅游警察队伍，率先建成“1+3”[2]旅游市场综合监管模式，旅游联合监管“一盘棋”格局基本形成。实施“旅游精准营销”行动，借力春晚效应，与脸书、英国广播公司等国内外知名媒体开展营销合作，开通“灵渠”号高铁旅游专列，飞行航线增至109条，通航城市达75个，机场旅客吞吐量近800万人次，铁路旅客发送量突破1500万人次，均创历史新高。

　　(二)聚焦实体经济，产业做强升级迈出新步伐

　　重振工业雄风成效初显。抓重点园区，新建标准厂房22.7万平方米、人才公寓(孵化器)16万平方米，园区承载能力显著提升。经开区新开工工业项目27个，完成投资21.3亿元，豪文学校等10个配套基础设施项目加快推进;高新区扩容提质取得积极成效，新建成的桂电校友产业基地入驻高科技企业23家，高新技术产业加快发展;高铁经济产业园总体规划基本完成，完成征地拆迁1万亩，引进重点项目6个，总投资25亿元;平乐工业集中区建设和招商引资成效显著，荔浦、灌阳分别引进荣事达、雅高控股等知名企业，全市三大园区“齐头并进”、各县工业集中区“多点支撑”的工业发展新格局初步形成。抓重大项目，华为产业合作区招商中心、展示中心、双创园(一期)和三金中药城等项目建成，莱茵生物完成搬迁，比亚迪新能源汽车等项目加快推进。抓政策引导，落实中央、自治区降本减负政策措施，出台加快桂林工业发展16条具体政策，发展环境持续优化。抓企业服务，坚持“市长服务日”等工作机制，建立完善非公经济服务平台，深入推进“惠企贷”业务，举办首届“桂林绿色制造”展示会，组织企业参加各类工业产品交易会，桂林产品市场影响力持续提升。

　　现代农业提质增效。持续实施现代特色农业“7+3”提升行动[3]，9个南方现代草地畜牧业项目加快推进，水果总产量达543万吨，灌阳“一季稻+再生稻”平均亩产再创世界纪录。加快现代特色农业示范区建设，新增自治区级(核心)示范区14个，获评自治区五星级示范区3个，各级示范区增至186个，数量居全区第一。大力实施农业品牌战略，新认证“三品一标”产品20个，龙脊梯田系统入选全球重要农业文化遗产，永福罗汉果入选首批中国特色农产品优势区名录，全州成为国家农产品质量安全县创建试点单位，永福龙江乡、灵川小平乐村成为全国一村一品示范村镇。积极培育新型农业经营主体，新增农民专业合作社552家、家庭农场135家、龙头企业37家。休闲农业继续走在全国前列，乡村旅游人数突破3000万人次，综合收入超50亿元。农民增收居全区前列。加强农业基础设施建设，完成永久基本农田划定工作，新增高效节水灌溉面积2.37万亩，荣获全国“平安农机”示范市。成功举办第十三届广西名特优农产品(桂林)交易会及桂林名特优新农产品上海、西安推介会，有效提升了我市农产品的美誉度。全国第三次农业普查取得阶段性成果。

　　服务业发展量质齐增。桂林创意产业园等3个集聚区成为自治区首批现代服务业集聚区。加快商贸服务业发展，叠彩万达广场等投入运营;桂林米粉速食保鲜技术实现突破，为大规模外销奠定了基础。健康养生、金融、会展等现代服务业加快发展，桂林国际智慧健康旅游产业园等项目加速推进，成功举办中国(桂林)国际健康旅游高端论坛，金融机构各项存贷款余额分别增长10.2%、15.5%。新兴服务业蓬勃发展，互联网和相关服务业营业收入增幅超80%，软件和信息技术服务业营业收入增幅超200%。“电商桂林”建设加快推进，电子商务交易总额增长23%，资源成为国家电子商务进农村综合示范县，我市获批总数增至6个，居全区前列。

　　(三)聚焦项目建设，基础设施攻坚取得新突破

　　建立市领导“一联三”工作责任制[4]，统筹推进重大项目889个，预计完成投资834亿元，增长10.4%。两江国际机场扩建工程加快建设;桂林动车所、西货运中心站投入使用;桂林至三江、资源至兴安高速公路建成通车，桂林至柳城、灌阳至平乐高速公路开工建设;永安关经水车至灌阳等国省干线公路升级改造加速推进，桂林汽车客运南站等项目进展顺利;平乐印山旅游码头一期工程投入使用，区域性综合交通枢纽地位更为凸显。斧子口水库基本建成，长塘水库项目前期工作有序推进。5个风电项目竣工投产，新增风电装机容量40万千瓦。电信普遍服务试点城市项目加快推进。通过发行企业债券、与金融机构加强战略合作等方式，融资近200亿元，有力保障了项目建设资金需求。

　　(四)聚焦协调发展，城乡建设展现新面貌

　　坚持新区老城双举并重、城乡一体统筹推进，新型城镇化建设成效显著，常住人口城镇化率达48.7%。加快建设临桂新区，投资发展大厦、建设大厦等工程完工，市民广场、山水公园建成开放，景观水系基本完工，复兴小学、桂林师专临桂校区投入使用，桂林中学临桂校区(一期)等工程竣工，旅游专线试验线、旅游综合医院等项目顺利推进，一座宜居宜业宜游的现代化新城加速崛起。加快疏解提升老城，全面推进“城市双修”[5]，芦笛路、甲山路铁路下穿通道建成通车，12座人行天桥主体完工，51条城市道路“白改黑”[6]全面完成，东二环路机动车道完成整修，王城片区等特色街区改造“书记工程”顺利推进，福隆园、塔山、新生街等片区改造加快实施，以项目带动片区改造模式获得普遍赞誉。着力提升城市管理水平，强化城市管理绩效考评，推进城市建筑垃圾公司化处置，老城区环卫机扫率达80%，环卫保洁市场化率达75%，完成拆违265万平方米，拆除违法户外广告牌7800多块，改造提升无物业管理小区100个，城市面貌明显改观。加快推进城乡一体化建设，全州、荔浦等7个国家和自治区级新型城镇化示范县、23个自治区百镇建设示范工程顺利推进，兴安溶江镇成为全国特色小镇。加快推进“美丽乡村”建设，“三改”工作[7]成效明显，31个传统村落保护发展示范村建设基本完成，200个现代特色生态宜居乡村示范村加快建设，灵川大桐木湾村成为全国生态文化村;建制村通客车率达91.8%、通公交率达39.1%，荔浦成为广西唯一的全国“四好农村路”示范县。

　　(五)聚焦改革创新，经济社会增添新活力

　　大力推动供给侧结构性改革，全面落实“三去一降一补”[8]五大重点任务，关停22家小造纸厂，全面取缔“地条钢”[9]，房地产去库存取得积极成效，直接融资占融资总额的比重超30%，为企业和社会减税清费27.59亿元。全面深化重点领域改革，漓江“三统”改革[10]加快推进，漓江旅游实施“四分四化”模式[11]，精华段游船全面提档升级;行政审批制度改革向纵深推进，市县两级行政审批事项精简率达55%;商事制度改革继续深化，在全区率先实现企业登记全程电子化覆盖，新增市场主体4.6万户，增长16.2 %;国企改革成效明显，原市级投融资公司和22家相关企业重组为8家投融资公司;金融体制改革加快推进，建立“4321”新型政银担合作关系[12]，新增“新三板”挂牌企业2家;农村综合改革全面深化，111个乡镇完成“多所合一”改革，农村土地承包经营权确权登记颁证、国有林场改革稳步推进;教育、科技和社会治理等领域改革持续推进。全面深化医疗改革，“五项制度”[13]建设成效显著，分级诊疗有序推进，家庭医生签约服务覆盖面不断扩大，持续开展医联体建设，灌阳医改模式得到国家层面充分肯定。开放水平进一步提升，内外资实际到位资金增长14.1%，进出口贸易总额增长11.8%。创新能力不断增强，国家可持续发展议程创新示范区申报工作取得重大进展，桂林国家农业科技园区通过验收。新增国家地方联合工程研究中心1家，桂林科技企业发展中心成为国家级小微企业创业创新示范基地，民华科技获评国家中小企业公共服务示范平台;获广西科学技术特别贡献奖1项、广西科技奖36项，福达集团获国家智能制造示范项目1项。高新技术企业保有量突破150家，三金药业获全区唯一国家工业企业质量标杆，光隆光电公司光电子芯片项目建成投产，填补国内空白。申请发明专利5296件，每万人口发明专利拥有量居全区前列，荔浦成为国家知识产权强县工程示范县。1人入选国家“千人计划”，新增“八桂学者”7人、广西特聘专家4人，选聘第二批“漓江学者”8人，新建广西院士工作站7家，新入站两院院士9人。

　　各位代表!创新引领发展，人才成就未来。让我们向为我市经济社会发展作出突出贡献的优秀人才，致以崇高的敬意和衷心的感谢!

　　(六)聚焦文化建设，城市软实力跃上新台阶

　　加强历史文化保护与利用，甑皮岩国家考古遗址公园(二期)建成开放，靖江王陵国家考古遗址公园考古挖掘清理工作基本完成，桂海碑林古代大型石刻展项目完工，靖江王府历史文化旅游休闲街区、非物质文化遗产展示馆等工程进展顺利，红军长征过桂北历史遗存发掘保护工作持续推进，完成一批文化标识设立和名人故居修缮，编纂《桂林历史文化大典》，出版《桂林雁山园》，历史文化遗产“散珠碎玉”得到系统性整理、完整性呈现、保护性利用。扎实推进文化惠民工程，展览馆新馆正式对外开放，建成村级公共服务中心963个，实现“村村有书屋”，广播、电视综合人口覆盖率分别达97.0%、98.5%，正阳东西巷“讲古堂”正式开讲，“百姓大舞台”“百姓大讲坛”“漓江之声”“周末大家乐”“桂林有戏”等群众性公益文化特色品牌影响力持续提升。与中国歌剧舞剧院联合创排的民族歌剧《刘三姐》首获国家艺术基金资助，方言话剧《龙隐居》获广西文艺创作铜鼓奖，纪录片《桂林古村落》被文化部收录，精品创作喜获丰收。加快发展文化产业，希宇文化创意产业园、漓江千古情等重大项目加速推进，桂林文体集团正式成立;力港网络销售收入突破7亿元，坤鹤“可可小爱”系列正能量短剧影响力进一步扩大，两家特色文化企业成为全区行业龙头，动漫产业继续领跑广西。

　　(七)聚焦漓江保护，生态环境实现新优化

　　坚持以国家生态文明先行示范区建设为抓手，加强环境保护和生态文明建设。严格落实环境保护“党政同责、一岗双责”，强化目标任务考核，全力推进中央环保督察反馈意见整改工作。构建漓江生态保护新格局，完善漓江风景名胜区生态保护长效管理机制，四级网格化监管体系不断健全;集中开展“四乱一脏”专项整治行动[14]，漓江流域21家关停采石场全部生态复绿，漓江市区段洲岛鱼餐馆及违法搭建全部拆除;漓江城市段污水集中治理基本完成，城市污水集中处理率超99%，污水直排漓江现象得到有效遏制。加强环境保护治理，强化大气污染防治，市区空气质量优良天数增至308天，是全区唯一实现正增长的城市，可吸入颗粒物(PM10)、细颗粒物(PM2.5)平均浓度值分别下降6.3%、6.4%;全面推行河长制，全市集中式饮用水水源地水质达标率100%，水环境质量居全区前列;土壤污染防治成效明显。加强生态城乡建设，扎实抓好生态公益林、天然林保护，植树造林26.6万亩，会仙喀斯特国家湿地公园试点建设通过国家验收，平乐狮子山跻身国家级森林公园，永福、恭城获广西园林城市称号，灵川、全州、阳朔、资源、恭城、临桂成为自治区级生态县(市、区)，生态创建工作保持全区领先。

　　(八)聚焦精准施策，脱贫攻坚取得新成果

　　整合各类扶贫资金39.2亿元，深入开展“七个一批”[15]“十大行动”[16]，全力以赴打好脱贫攻坚战。龙胜、资源、灌阳三个扶贫开发重点县脱贫攻坚工作成效显著，龙胜获批国家健康扶贫示范县;资源以有机产业助推精准扶贫，跻身国家有机产品认证示范县;灌阳创新推出“电商扶贫直通车”模式。出台《桂林市“十三五”农村脱贫攻坚重点项目资金以奖代补实施办法》，开展基础设施建设大会战，新建改建贫困村公路1440条，实施农村电网改造项目62个。大力发展果蔬种植、禽畜养殖等产业，受益贫困户达40752户。开工建设易地扶贫搬迁点33个，累计搬迁入住21771人。发放扶贫贷款13.4亿元，3.31万贫困户获得小额信贷，工作经验全区推广。累计发放教育扶贫资金1.57亿元，惠及学生24.5万人次。提高建档立卡户参合人员普通住院费用补偿10个百分点，大病保险补偿起付线从8000元降低至4000元。与肇庆市扶贫协作取得积极进展。村级集体经济组织“造血”功能不断增强，贫困地区呈现脱贫提速、发展提效、民生提质的良好局面。

　　(九)聚焦民生需求，人民群众满意度得到新提升

　　把保障和改善民生作为政府一切工作的出发点和落脚点，民生领域支出占一般公共预算支出总额的79.1%，自治区层面十大类33项、市级层面十大类34项为民办实事项目全面完成。扎实推动“大众创业、万众创新”，城镇新增就业6.5万人，农村劳动力转移就业新增7.63万人次，城镇登记失业率2.4 %。健全社会保障体系，“五险”[17]参保人数716万人次，发放城乡低保5.95亿元，开工建设棚户区改造住房10312套，基本建成保障性住房12451套，农村危房改造新开工14504户。办好公平优质教育，新续建中小学47所，永福通过国家义务教育基本均衡县认定，兴安、叠彩等9个县区通过自治区评估，学前教育三年毛入园率、九年义务教育巩固率、高中阶段教育毛入学率分别达85.0%、97.5%、91.3%，桂林高校集聚区建设取得重要进展。深化健康桂林建设，疾病防控工作扎实有效，卫计资源整合走在全国前列，中医壮瑶医药不断发展，率先在全区发行居民健康卡，计划生育服务管理水平不断提高，秀峰社区卫生服务中心荣获全国卫生计生系统先进集体，象山平山、叠彩北门社区卫生服务中心成为全国百强社区卫生服务中心。扎实开展全民健身活动，社区全民健身设施覆盖率达80%，名列全区前茅。深入推进平安桂林建设，严厉打击“两抢一盗”、黄赌毒、电信诈骗等犯罪活动，社会治安防控体系进一步完善，连续5次被评为全国社会治安综合治理优秀市，连续3次获得“长安”杯。民族团结进步创建工作扎实推进，恭城成为全国民族团结进步创建示范区。安全生产形势持续稳定，食品药品监管成效明显，依法调处化解各类纠纷，社会保持和谐稳定。

　　一年来，我们深入学习贯彻党的十九大精神，全力推进“两学一做”学习教育常态化制度化，政府治理能力进一步提升。坚持依法行政，健全重大行政决策机制，实施重大决策预公开，依法接受市人大及其常委会的监督，自觉接受市政协的民主监督，人大代表建议和政协提案办结率达100%。优化政务服务，落实行政审批“三集中三到位”[18]制度，完善“马上办”工作机制，办理时限从法定平均时间22.53天提速至9.1天，办理提速率达83.1%，按时办结率100%。切实履行全面从严治党主体责任和党风廉政建设“一岗双责”，认真贯彻落实中央八项规定精神和国务院“约法三章”要求，加强党风廉政建设和反腐败工作，抓好巡视“回头看”反馈意见整改，加强重点领域的审计监督和行政监察，查处违反八项规定精神问题291起，处理338人，全区扶贫领域监督执纪问责工作推进会在桂林召开。健全厉行节约长效机制，公开部门预算和“三公”经费预决算，“三公”经费同比下降19.2%，作风建设取得明显成效。

　　一年来，国防教育深入开展，国防和后备力量建设得到加强，人民防空工作扎实推进，民族宗教、外侨、对台工作、编制、人事、统计、质监、物价、接待、机关事务、档案、供销、保密、口岸、气象、水文、测绘、地方志、检验检疫、防震减灾、决策咨询、社会科学、残疾人、公共机构节能等工作取得新进步，中直、区直驻桂林单位取得新成绩，工会、共青团、妇联等群团组织在经济社会发展中发挥了重要作用。

　　各位代表!看似寻常最奇崛，成如容易却艰辛。这些成绩，是在经济下行压力持续加大的形势下取得的，是在注重优化经济结构、提高质量效益的情况下取得的，是在推进供给侧结构性改革、加快淘汰落后产能的环境下取得的。这是市委总揽全局、科学决策的结果，是市人大及其常委会和市政协监督支持的结果，是全市各族干部群众团结拼搏、开拓进取的结果。在此，我代表市人民政府，向全市各族人民，向人大代表、政协委员，向各民主党派、工商联、无党派人士、各人民团体和各界人士，向离退休的老领导、老同志，向中央和自治区驻桂林单位，向驻桂林部队和武警官兵，向所有关心支持桂林发展的海内外朋友，致以崇高的敬意和衷心的感谢!

　　在充分肯定成绩的同时，我们也清醒地看到，我市发展还面临不少问题和困难，主要表现在：经济总量小，人均水平低。产业发展不充分，工业不大不强，结构不优，效益不高;服务业企业实力总体偏弱，核心竞争力有待进一步提升;名牌产品数量少，影响力不强。县区之间、城乡之间发展不平衡，民生领域尚有短板，脱贫攻坚任务艰巨，公共服务能力建设还有较大提升空间，等等。我们要正视困难，直面矛盾，进一步解放思想、敢作善为、精准施策，持续开创经济社会发展新局面。

　　二、2018年工作总体要求和目标任务

　　2018年是贯彻党的十九大精神的开局之年，是改革开放40周年，是自治区成立60周年，是决胜桂林“两个建成”的关键一年。我们既要看到形势的积极方面，坚定信心，也要看到形势的复杂性，增强忧患意识，兢兢业业、扎扎实实做好各项工作。

　　今年政府工作的总体思路是：全面贯彻党的十九大精神，以习近平新时代中国特色社会主义思想为指导，围绕中央、自治区经济工作会议及市委五届四次全会决策部署，坚持稳中求进工作总基调，坚持新发展理念，紧扣社会主要矛盾变化，按照高质量发展要求，以推进供给侧结构性改革为主线，坚持桂林国际旅游胜地建设“一本蓝图绘到底”，围绕“加快建设新城、疏解提升老城、产业融合发展、城乡协调推进、生态文化相融、富裕和谐桂林”的总体要求，全面做好稳增长、促改革、调结构、惠民生、防风险各项工作，着力构建现代化经济体系，坚决打好精准脱贫攻坚战，加强和改善民生，促进经济社会持续健康发展，为决胜“两个建成”目标奠定坚实基础。

　　今年经济社会发展的主要预期目标是：地区生产总值增长7%-7.5%，规模工业增加值增长6.8%-7.5%，财政收入增长6.5%，固定资产投资增长12%，社会消费品零售总额增长10.5%，城镇居民人均可支配收入增长8.5%，农村居民人均可支配收入增长12%，居民消费价格涨幅控制在3%左右，城镇登记失业率控制在4.5%以内，常住人口城镇化率达50%。上述目标，体现了高质量发展和稳中求进的要求，考虑了各县区、各行业的发展实际，兼顾了需要与可能。

　　实现上述目标，重点要抓好以下工作：

　　(一)坚持国际标准，着力推进国际旅游胜地建设

　　持续推进世界一流旅游目的地建设。推进旅游产品国际化和品牌化，积极引进一批国际品牌酒店、休闲产品、购物中心，加速形成国际品牌聚集效应;加快打造龙脊梯田、动感天湖、资源八角寨、兴安灵渠、猫儿山养生度假区等世界级旅游精品;加快推进万达文化旅游城等重大项目，开工建设复星桂林健康养生文旅小镇、龙光国际健康休闲养老小镇、七星体育休闲生态度假区，推动阳朔乌布小镇与洲际酒店等一批国际性品牌酒店群建成运营，不断完善符合国际化要求的旅游产品体系。推进旅游服务标准化，提供与国际接轨的餐饮住宿、旅游购物、导游等服务，加快建设一批具有国际品质的旅游服务中心，重点打造市区一级旅游集散中心和阳朔、兴安、龙胜二级旅游集散中心，完善景区道路多语种标识系统，强化旅游管理服务人才引进和培养。推进旅游基础设施便利化，完成两江国际机场扩建工程，积极培育新航线，扶持壮大桂林航空;加快打造大桂林生态休闲旅游精品线路，全力推进龙胜生态旅游大环线公路等项目建设;完善旅游交通换乘系统，建立无缝衔接的交通网络。加快推进“一部手机游桂林”。上半年，通过政府引导、市场运作，整合现有信息化成果，按照“硬件+软件+平台+服务”模式，重点建设全市统一的旅游大数据中心、旅游服务平台、旅游管理平台，实现手机查询、预定、支付、评价、宣传、监管等功能，提升旅游便捷性与舒适度。

　　全力推进旅游创新发展。深入实施“旅游+”战略，推动旅游与相关产业融合发展，围绕“吃住行游购娱”“商养学闲情奇”十二要素，重点培育乡村旅游、体验旅游、研学旅游、体育旅游、健康养生游等新业态，不断完善旅游产业体系。大力推进旅游“双创”工作，抓好荔浦、资源、恭城、灵川、雁山、灌阳等县区广西特色旅游名县创建工作，加快阳朔、兴安、龙胜、恭城、雁山、灌阳等县区国家全域旅游示范区创建步伐，持续推进26个特色旅游小镇建设，形成开放、共享、精细、高效的全域旅游发展新格局。加快市级旅游资源整合，基本完成旅游企业重组，优化市区旅游大景区管理模式，着力打造旅游“旗舰企业”“规模景区”“特色产品”。不断创新旅游管理体制，加快桂林旅游产业研究院建设，持续推进“1+3”旅游市场综合监管，重拳整治旅游市场秩序，进一步优化旅游发展环境。

　　持续推进旅游区域合作。继续办好联合国世界旅游组织/亚太旅游协会旅游趋势与展望国际论坛、中国—东盟博览会旅游展、桂林国际山水文化旅游节、环广西公路自行车世界巡回赛(桂林段)、桂林国际马拉松赛、中国·阳朔国际山地越野赛、中国(桂林)国际健康旅游高端论坛等重大节事、赛事活动，积极策划一批新的国际赛事，吸引一批跨国公司国际年会在桂林召开，争取世界旅游组织每年通过桂林论坛发布世界旅游趋势报告，引领世界旅游发展潮流。加强国际营销与推介，突出打响桂林世界自然遗产地、国家历史文化名城品牌，吸引更多境内外游客。力争旅游人数增长20%，旅游总消费增长25%。

　　促进服务业优质高效发展。加快发展健康养生产业，建设国家健康旅游示范基地，落实《桂林市健康旅游产业发展规划》和《桂林国家健康旅游示范基地建设实施方案》，按照“一核两线、多点辐射”思路，强化招商引资和重点项目建设，促进健康服务和旅游产业深度融合，大力发展高端医疗、康复保健、休闲养生等新业态，提高桂林健康旅游产业的竞争力，打造“漓水青山，养生桂林”城市品牌。促进商贸服务业集聚发展，老城区重点打造以万福广场为中心的南部商圈，以叠彩、七星万达广场为中心的北部和东部商圈，以华润琴潭片区为中心的西部商圈，以王城片区为中心的历史文化休闲旅游中部商圈;新区加快培育以海吉星为重点的新商圈。持续推进“电商桂林”建设，大力促进“电子商务进农村”，继续开展“桂林网购节”等主题活动，不断扩大社区智能快递终端覆盖面。大力发展金融、信息服务、现代物流、商务会展、体育等产业，建立完善“四上”企业[19]培育机制，力争新增规模以上服务业企业150家。进一步对38个服务业集聚区进行系统梳理，分类加速推进，着力打造区域性现代服务业中心城市和面向东盟的现代服务业创新发展示范区。

　　(二)重振桂林工业雄风，着力构建现代产业体系

　　狠抓生产要素集中。突出抓好工业园区基础设施建设，推动土地、金融、人才、技术等要素向园区集中。加快园区土地收储和标准厂房建设，新建50万平方米标准厂房、20万平方米人才公寓(孵化器)，为项目入驻创造良好条件。继续抓好华为产业合作区基础设施建设，推动入园企业扩大投资，建成华为双创园、数据中心(一期)等项目，建成中兴通讯产业园招商中心、展示中心、5万平方米标准厂房，加快比亚迪新能源客车生产基地建设，积极推动手机项目落地，力争八加一大型医疗设备生产线建成投产。推动金融资源向园区集中，引导银行、保险、担保等金融机构入园设立分支机构，为企业提供便捷的金融服务。加强招才引智，完善园区住房、教育、医疗、商业等配套服务设施，营造一流的工作生活环境，吸引一流人才到园区就业创业。实施科技入园工程，推动科技经费向园区倾斜，引导科研机构向园区集聚，支持驻桂林高校院所到园区创办园中园，加速科研成果转化，提升园区自主创新能力。加快发展水路运输，重点推进平乐珠子洲客货综合港、永福洛清江等水运项目规划建设;经开区、高铁经济产业园着力引进一批跨区域大型物流企业入驻，进一步降低园区企业物流成本。

　　狠抓产业集聚发展。围绕电子信息、医药及生物制品、先进装备制造、生态食品四大优势产业和新能源、新材料、新一代信息技术、节能环保等新兴产业，大力引进行业龙头企业和关联企业，不断完善产业链，打造产业集群。依托华为、中兴等知名企业，推动电子信息产业加快发展。依托比亚迪、桂林客车、福达等大型企业，打造桂林新能源客车生产基地。依托光隆光电等高科技企业，打造桂林“微芯谷”。依托力源粮油、漓泉啤酒、莱茵生物、三金药业等企业，加快建成全国知名的生态绿色食品和大健康产业基地。积极发展固体动力电池、石墨烯、新型传感器等核心产业和关联产业。依托现代科技改造提升传统产业。推动军民融合深度发展，加快推进军民融合产业基地规划建设。

　　狠抓数字经济发展。深入贯彻国家大数据战略，大力推动互联网、大数据、人工智能与实体经济深度融合。持续推进“宽带桂林”建设，完善通信网络等信息基础设施。加快建设大数据中心、云计算中心，带动软件、物联网及通讯设备、智能终端等关联产业快速发展。尽快启动华为、中兴桂林研究基地建设。推动数据资源整合应用，建设健康医疗大数据中心，大力发展共享经济。继续推进两化融合贯标对标工作，鼓励企业发展数字化车间和智能工厂，实施机器换人、个性化定制等新模式，加快推进智能制造。

　　狠抓企业培育服务。坚持“市长服务日”等工作机制，主动融入广西非公经济服务平台，全面落实中央、自治区及我市加快工业发展的政策措施，不断优化实体经济发展环境，打造一批年产值超10亿、20亿、30亿的行业小巨人。探索建立工业企业分类评价体系，实行差别化管理。抓好重点骨干企业培育，加快打造桂林特色产业集群。抓好规模企业培育，争取实现新上规入统企业60家以上。抓好小微企业扶持，发挥其协作配套功能。抓好困难企业帮扶，为动力转换赢得时间。抓好成长型、创新型企业扶持，培育研发与营销两端创新型企业，支持智神、海威等科技型“瞪羚企业”加快发展。抓好桂林品牌产品本地应用，推动本地企业与国内外大型知名企业产销对接，不断拓展产品市场。抓好老城区工业企业搬迁改造，推动企业实现产值效益大提升。

　　(三)实施乡村振兴战略，着力推进农业农村现代化

　　扎实推进现代特色农业建设。贯彻全区现代特色农业“10+3”提升行动荔浦会议精神。实施现代特色农业示范区增点扩面提质升级三年行动，加大创建整合力度，打造一批有规模、上档次的示范区，努力实现县县有自治区级(核心)示范区、每个乡镇建成一个自治区乡级以上示范区，积极创建国家级、自治区级特色农产品优势区。推动农村一二三产融合发展，大力发展农产品加工业和物流业，构建现代化大农业全产业链;做好“农业+”文章，打造一批田园综合体，创建桂林休闲农业国际旅游示范区。持续推进品牌建设，积极培育农产品地理商标、区域公用、企业、产品等品牌，力争新增认证“三品一标”产品30个以上。办好第十五届广西名特优农产品交易会，在全国主要城市举办特色农产品推介会。狠抓农产品标准化生产、质量安全监管，做好柑橘黄龙病综合防治工作，加强畜禽养殖废弃物资源化利用，推广种养结合型生态循环农业技术模式，力争65%以上规模养殖场实现生态养殖，推动质量兴农、绿色兴农。大力提高农业综合生产能力。继续推进高标准农田建设，开展粮食绿色高产创建，巩固提升粮食产能。继续实施高效节水灌溉、中小河流治理等水利设施建设项目，充分发挥气象、水文等服务保障作用，提高农业防灾减灾能力。推动农业机械化全程、高效发展，力争农业耕种收综合机械化水平达60%以上。发挥好桂林国家农业科技园区示范作用，推进基层农技推广体制机制创新。实施新型职业农民培育整村推进工程，鼓励社会各界人士投身乡村建设。

　　持续开展“美丽桂林”乡村建设。完成宜居乡村、启动幸福乡村建设。深入推进产业富民、服务惠民、基础便民专项活动，全面完成“三改”重点任务，实现农村清洁厨房普及率60%以上、无害化卫生厕所普及率90%以上、人畜混居改造完成率50%以上，实现行政村基本公共服务全覆盖、群众办事不出村。加快“四好农村路”建设，新增村际联网路200公里以上。创建一批国家级美丽宜居小镇、宜居村庄，建设88个宜居乡村示范村，建成第三批18个传统村落保护发展示范村，持续改善农村人居环境。

　　不断深化农村改革。推进农业供给侧结构性改革，探索“三权分置”[20]多种实现形式，完成农村土地承包经营权确权登记颁证工作，加快荔浦集体土地流转服务平台建设试点工作。培育新型农业主体，引导和支持种养大户、家庭农场、农民专业合作社、龙头企业发展壮大，提升辐射带动功能。统筹推进小型水利工程管理体制、国有林场、供销合作社、农村金融等重点领域改革，激发农业农村发展新动能。

　　(四)实施项目带动，着力增强发展后劲

　　掀起项目建设新高潮。实施市级统筹推进重大项目969个，完成投资917亿元。强力推进十大重中之重项目和市领导跟踪服务的62个重大项目建设，特别要抓好万达、华为、比亚迪等重大产业项目建设。确保完成桂林汽车客运南站等一批重大项目;加快推进灌阳至平乐、贺州至巴马(桂林段)、荔浦至玉林、桂林至柳城等一批在建项目，争取阳朔至鹿寨高速公路复工。启动市区及与各县之间快速交通系统规划建设。全面建成桂林防洪及漓江补水枢纽工程，推进长塘水库前期工作，加快巴江口船闸改扩能工程建设。

　　激发民间投资活力。坚持以市场的理念、市场的手段、良好的服务，促进民间投资较快增长。支持民间资本以独资、合资、控股、参股、合作等方式依法开展各种投资活动;对于社会资本不愿进入的“小散”项目，通过“捆绑打包”“上下游串联”等方式激发社会投资意愿。

　　强化项目服务保障。做好项目包装策划，围绕胜地建设，超前谋划、科学编制三年滚动投资计划。加强项目动态管理，让具备条件的项目加快推进，让久推不动的项目及时退出。坚持“五个一”工作机制[21]，完善“1+4”会议制度[22]，形成项目推进的强大合力。整合不动产登记业务，实现“一个窗口受理、一站式服务”，提升不动产登记效率;加快购进储备占补指标，全面保障项目建设用地;大力破解征地拆迁难题，探索开发区村庄原地改造模式，打造与开发区配套的现代金融小镇、智慧小镇、特色旅游小镇。

　　(五)打造品位休闲之都，着力推进新型城镇化建设

　　加快完善临桂新区功能。牢固树立城市品牌意识，按照全国文明城市标准，规划、建设和管理新区，提升新区品质。大力推进机场路以北、万平路以南片区规划建设。围绕入驻入居，完成金融大厦、广电大厦、桂林日报社传媒中心等项目建设。加快推进国际会展中心、科技馆、规划馆等项目。开工建设西城大道南延长线等市政项目，加快旅游专线试验线建设，扎实推进北区水系工程。力争桂林师专全部搬迁入驻临桂校区，桂林中学临桂校区、旅游综合医院投入使用，加快推进崇文小学等学校建设。规划建设一批专业市场，建成枫林等农贸市场，推进吾悦广场等城市综合体建设，引进酒店、商业、教育、医疗等方面的知名品牌入驻，加快打造产业新城、宜居新城。

　　加快疏解提升老城。继续实施“城市双修”8大类39个示范项目，加快修复城市设施、空间环境、景观风貌。实施畅通缓堵工程，打通一批城市断头路，推进主要道路路口渠化改造，建成15座人行天桥，新建和改造20个以上立体停车场。推动城市业态提升，抓好城区产业“退二进三”，带动休闲旅游、文化创意、楼宇经济、拍卖等产业发展。加大城市改造力度，着力推进城区北大门片区建设，加快灵川县与桂林同城化建设步伐，加速打造漓江城市段中心区域休闲旅游示范带，推动龙船坪等特色街区改造提升，加快福隆园、塔山、新生街、华润琴潭等片区改造，抓好洲岛、漓江和小东江两岸环境整治。持续推进全国文明城市创建活动，巩固提升已有成果，将金点子、城市立面定期清洗等有效做法上升为制度标准，推动城市管理长效化、精细化、智能化。迎接新一轮国家卫生城市复审。大力宣传实施《桂林市城市市容和环境卫生管理条例》，完善城市管理市场运作机制，进一步做好“人机清扫”巡回保洁等工作。新增20-30个垃圾分类试点小区，实施100个无物业管理小区改造。拆除城市建成区违法建设254万平方米，坚决遏止新增违法建设。继续开展占道经营整治等专项活动，完成主次干道“白改黑”并向小街小巷延伸，实施绿化、花化、彩化等工程，创造更加优美的城市环境。

　　加快推进县域经济发展。突出产业发展，落实和完善促进县域经济发展政策，每县力争引进1-2个对财政起支撑作用的重大产业项目，培育1-2个特色产业集群。实施项目带动，谋划一批发展潜力大、带动作用强的项目进入自治区三年滚动计划，夯实县域经济发展基础。强化基础支撑，强力推进县域基础设施能力提升三年行动计划，推动“路、水、电、网、气、园”等建设，提升县域经济发展支撑能力。完善县域经济综合评价考核指标体系，发挥评价考核的导向作用。继续实施大县城战略，按照城市标准进行规划建设管理。落实扩权强县、扩权强镇改革，加快荔浦、全州撤县改市步伐，推动符合条件的乡撤乡改镇。突出抓好7个国家和自治区级新型城镇化示范县、第三批7个自治区百镇建设示范工程建设，完成第三批16个、启动第四批13个新型城镇化示范乡镇“书记工程”，着力打造县域经济社会发展次中心。

　　(六)坚持生态立市，着力推进生态文明示范区建设

　　强化漓江科学保护利用。加快编制漓江风景名胜区详细规划，推动出台《漓江风景名胜区管理条例》。继续强化漓江流域生态环境综合整治，抓好漓江城市段截污工程(三期)，实现漓江流域市区段截污管网全覆盖;加强漓江水源林保护、生态修复、水资源调度，强化网格化管理和日常巡查，巩固采石场整治、畜禽养殖场禁限养、网箱养鱼清理成果，深入推进“四乱一脏”专项整治。全力推进漓江旅游码头提升改造、漓江城市段游船提档升级。推进漓江城市段洲岛自然生态公园项目，完成漓江城市段东岸慢行步道建设，加快形成漓江旅游新景观。

　　持续抓好环境污染防治。全面完成自治区对我市能源消费总量和强度“双控”目标责任制考核。扎实开展第二次全国污染源普查，抓好中央环保督察反馈意见的整改工作。继续实行双目标联控，健全网格化管理体系，力争可吸入颗粒物(PM10)浓度降至每立方米66微克以下，细颗粒物(PM2.5)浓度降至每立方米44微克以下，空气优良率达85%以上，坚决打赢蓝天保卫战。强化水污染防治，全面推行河长制，进一步加强河流水库管理保护和水环境治理，加强饮用水水源地保护和备用水源地建设，加速推进城北水厂二期工程，完成市区污水厂提标改造，基本消除城市黑臭水体。强化土壤污染管控和修复，保持全市土壤环境质量总体稳定。

　　加大生态系统保护力度。扎实推进生态文明建设，开展国土绿化行动，完成营造林17万亩，严禁毁林种果，加强野生动物保护。大力推进国家湿地公园试点和自然保护区建设。抓好国家生态园林城市、国家森林城市、国家园林县城创建工作，加快城市山体、河流、湿地、植被等生态修复。持续推进生态县、生态乡镇、生态村创建工作。

　　各位代表!生态文明建设功在当代、利在千秋。我们要始终牢记习近平总书记“一定要保护好桂林山水”的重托，确保桂林蓝天常驻、青山常在、碧水长流!

　　(七)持续深化改革创新，着力推动高质量发展

　　深化供给侧结构性改革。大力破除无效供给，全面完成自治区下达的化解过剩产能任务，严禁新增产能过剩项目。着力化解非住宅商品房库存压力，积极发展住房租赁市场。着力防控金融风险，健全政府性债务管理长效机制，加强民间融资、互联网金融、企业债券等风险防控。继续清理涉企收费，大力降低实体经济成本。

　　着力提升创新驱动能力。积极创建国家可持续发展议程创新示范区，创新生态保护、科技成果转化、多元化投融资等机制，实施自然景观资源保育、生态产业创新发展、创新驱动能力支撑等行动，促进生态保护与绿色产业协调发展，支持恭城等县区率先实施可持续创新发展，为世界同类地区提供现实样板和典型经验。深入推进桂林自治区级自主创新示范区建设。促进发明专利量质提升。大力培育具有创新能力的排头兵企业，打造一批年产值3亿元以上的科技型企业，新增高新技术企业30家以上、自治区级科技平台5家以上。实行柔性引才政策，加快创建高层次人才一站式服务平台，加强海内外高端人才创业创新示范基地建设，力争引进创新型高层次人才50人以上。

　　实施质量强市战略。大力弘扬“一流山水，一流质量”的桂林质量精神，建立健全高质量发展的考评体系，持续开展质量提升行动，积极创建“全国质量强市示范城市”。加大品牌培育和保护力度，打造品牌形象突出、产品质量一流的现代企业，为高质量发展奠定坚实基础。

　　持续推进重点领域改革。深化行政审批、“放管服”、“多证合一”等制度改革，激发市场主体活力。继续推进漓江“三统”改革，大力推行漓江旅游经营“四分四化”新模式。深化投融资体制改革，推进市城投集团组建工作。深入推进园区管理体制、城市管理执法体制以及文化、教育、卫生、科技、脱贫攻坚等民生领域改革。深化统计管理体制改革，做好全国第四次经济普查和投入产出调查工作。

　　加大国企改革力度。筛选一批条件较好的国有企业，通过整合优质资源、注入优质资产等方式，将其打造成资产超千亿元企业。创新监管方式，加快完善以国有资产保值增值为核心的考评体系，激发企业发展的内生动力。切实发挥国有企业强大的融资功能，以及在重大项目建设、产业转型升级方面的带动作用，使其成为全市经济发展的稳定器和压舱石，成为贯彻落实市委市政府战略意图、引领全市经济社会发展的生力军。

　　构建开放新格局。积极融入“一带一路”建设，深入对接自治区“三大定位”，推进与高铁沿线及周边城市的产业互动发展。瞄准国内外500强、民企100强企业开展精准招商，力争招商引资到位资金增长8%以上，实际利用外资超5.5亿美元。加强与国外友好城市交流合作。实现外贸进出口总额稳定增长。

　　(八)建设文化强市，着力推动文化繁荣兴盛

　　保护传承城市文脉。深入实施“寻找桂林文化的力量，挖掘桂林文化的价值”工程，传承中华优秀传统文化，推动桂林文化创造性转化、创新性发展。实施重点文物遗址保护修复工程，完成王城片区历史文化旅游街区改造，加快国家考古遗址公园建设，着力推动灵渠申报世界文化遗产和世界灌溉工程遗产，加强古桂柳运河和会仙湿地保护开发，继续抓好红军长征过桂北等文物保护利用及名人故居、古迹修缮开放等工作，推进第三批文化标识建设。持续抓好《桂林历史文化大典》等编纂工作。组织开展文艺进校园、非遗展演展示等活动，形成保护文化遗产的良好社会氛围，彰显历史文化名城的永久魅力。

　　繁荣发展文化事业。强力推进乡镇广播电视无线发射台(站)、村级公共服务中心建设和农家书屋补充更新，持续开展送文艺、送电影下乡活动，继续推动图书馆、美术馆、展览馆、文化馆(站)等公共文化设施免费开放，进一步丰富“百姓大舞台”“百姓大讲坛”“漓江之声”“周末大家乐”“桂林有戏”等群众性公益文化特色品牌内涵，打通公共文化服务“最后一公里”。开展深入生活、扎根人民的主题实践活动，创作更多的文化精品，抒写桂林人民的美好生活。

　　提升文化产业竞争力。创新“文化+”发展模式，加快发展动漫游戏、创意设计、网络文化等新型文化业态，培育文化产业新亮点。支持重点文化企业做大做强，进一步提升力港动漫游戏、“可可小爱”等品牌影响力，争创1-2家自治区级以上文化产业示范基地。持续推进高新区文化创意产业园、希宇文化创意产业园、歌仙刘三姐、漓江千古情、象山景区实景演出等项目建设，争创国家级文化产业示范园区。实施文化走出去战略，鼓励动漫、演艺、美术等产业进入欧美、东南亚市场，提升桂林文化国际影响力。

　　(九)打好精准脱贫攻坚战，着力保障和改善民生

　　坚决打好脱贫攻坚战。坚持把脱贫攻坚作为最大政治责任、最大民生工程、最大发展机遇，全面深入实施精准扶贫、精准脱贫“七个一批“十大行动”，以钉钉子精神持续打好脱贫攻坚战。大力推进产业脱贫，培育一批能带动贫困户长期稳定增收的特色种养优势产业，积极发展旅游、电商等扶贫新业态。完善村民合作社体制机制，全力推进贫困村集体经济发展，确保每个贫困村集体经济收入超3万元。强力实施易地扶贫搬迁，完成7900人以上搬迁任务。抓好金融扶贫，加大涉农资金整合力度，扎实开展扶贫小额信贷工作。全力推进健康扶贫行动，切实减轻贫困人口看病治病负担。继续推进贫困村电网、道路、宽带建设，加快补齐基础设施短板。抓实定点帮扶和结对帮扶工作，统筹抓好教育扶贫、低保兜底、转移就业等工作。严格执行“一把手”脱贫攻坚责任制，抓好扶贫开发成效考核和实绩考核，切实增强脱贫实效。持续开展扶贫领域腐败和作风问题专项整治，确保脱贫攻坚风清气正。

　　抓好就业和社会保障。完善落实就业创业政策，大力开展职业技能培训，注重解决结构性就业矛盾，实现城镇新增就业5万人，城镇失业人员再就业1.47万人次以上，农村劳动力转移就业7万人次以上。健全社会保障体系，大力实施全民参保计划，深入推进医保支付方式改革，降低个人医保自费比例，完善统一的城乡居民基本医疗保险和大病保险制度。加大农村低保制度与扶贫开发政策的有效衔接，提高农村低保保障标准和补助水平。

　　协调推进社会事业。优先发展教育，探索教育管理体制改革，实施第三期学前教育行动计划，力争实现乡镇公办中心幼儿园全覆盖，优化中小学校布局，推动城乡义务教育一体化发展，做好国家县域义务教育均衡发展督导评估验收，加快普及高中阶段教育，继续做好中职学校布局调整及专业结构优化，完成市卫校入驻雁山校区。持续推进桂林高校集聚区建设，加快实施雁山大学集中区中心环线、芳香东路等项目建设，大力支持高校创造良好环境，引进各学科领军人才;不断完善产学研合作机制，积极构建产业技术创新战略联盟，推动高校与地方经济社会深度融合发展;推动桂林师专“升本”。积极发展民办教育。落实《健康桂林2030规划》，扎实推进健康桂林建设。加强公共卫生工作，提高疾病防控能力，不断提升基层医疗卫生机构服务水平;推进市级医疗资源整合，加快中医药事业发展;促进生育政策和相关经济社会政策配套衔接。创新食品药品监管模式，实施食品监管工程，大力推进广西食品安全示范城市、示范县创建工作。开展全民健身活动，全力打造城区15分钟全民健身圈。

　　加强和创新社会治理。扎实开展民族团结进步创建活动。深入推进法治桂林、平安桂林建设，实施“雪亮”工程，大力推进天网工程建设，健全立体化社会治安防控体系，依法打击和惩治黄赌毒、黑拐骗等违法犯罪活动。完善突发事件应急处置机制，做好信访维稳和矛盾化解工作。完善安全生产责任制，坚决遏制重特大安全事故，提升防灾减灾救灾能力。做好人民防空工作。扎实开展“扫黄打非”工作。加强社区治理体系建设，实现政府治理和社会调节、居民自治良性互动。

　　深入开展国防教育，加强国防动员和后备力量建设，加大双拥创建工作力度，让军人成为全社会尊崇的职业。

　　竭诚办好民生实事。继续实施为民办实事工程，确保完成自治区下达的重点民生项目任务。积极筹措资金，实施教育、文化、卫生、基础设施等十大类40个为民办实事项目，解决群众普遍关心的热点难点问题。

　　各位代表!“两个建成”的目标任务光荣而艰巨，人民群众对美好生活的向往现实而热切。新时代新形势对政府工作提出了新要求，我们必须以时不我待的紧迫感、夙夜在公的使命感，敢于打破常规，严格依法履职，诚心为民服务，通过一项项任务的完成、一个个项目的实施、一件件实事的落实，回应群众的关切与期盼，赢得人民的支持和拥护!

　　一要坚定政治方向。坚定不移落实“党领导一切”的根本原则，牢固树立“四个意识”，坚定维护以习近平同志为核心的党中央权威和集中统一领导，认真贯彻落实自治区党委、政府的决策部署，坚决维护市委总揽全局、协调各方的领导作用，以实际行动确保党的政令畅通。

　　二要严格依法行政。加强法治政府建设，严格按照法定权限和程序行使权力、履行职责。依法接受市人大及其常委会的监督，自觉接受市政协的民主监督，加强政府协商，密切与各民主党派、工商联和各人民团体、社会各界人士的沟通联系，认真办理市人大代表建议、市政协提案。完善政府议事规则和决策程序，进一步推进决策、执行、管理、服务结果公开，主动接受舆论监督和群众监督。

　　三要坚持高效施政。牢固树立“实干就是能力、落实才是水平”的理念，加快转变政府职能。坚持实行市长碰头会议制度、市领导定期坐班制度，集中解决政务问题。坚持领导干部调查研究制度，深入基层、深入群众，了解实情、解决问题。进一步强化机关效能建设，持续整治“为官不为”“推绕拖”等不良作风。落实容错纠错机制，旗帜鲜明为勇于改革创新者鼓劲撑腰。

　　四要坚持从严治政。严格落实管党治政的政治责任、主体责任，始终把全面从严治党要求落实到政府工作各领域和全过程。精心组织“不忘初心，牢记使命”主题教育，深入推进“两学一做”学习教育常态化制度化，严格落实中央八项规定精神，扎实开展形式主义和官僚主义专项整治，坚决防止“四风”问题反弹。持续推进廉洁政府建设，不断增强政治定力、纪律定力、道德定力、抵腐定力，始终做到公正用权、谨慎用权、依法用权，以实际行动树立政府的良好形象。

　　各位代表!新时代要有新气象，更要有新作为。让我们更加紧密地团结在以习近平同志为核心的党中央周围，高举习近平新时代中国特色社会主义思想伟大旗帜，在自治区党委、政府和市委的领导下，不忘初心，牢记使命，以永不懈怠的精神状态、一往无前的奋斗姿态，继续朝着“两个建成”的宏伟目标奋勇前进!
